# Development of Therapeutic Alliance and Social Presence in a Digital Intervention for Pediatric Concussion: Qualitative Exploratory Study

**DOI:** 10.2196/49133

**Published:** 2024-03-22

**Authors:** Kiarah M K O'Kane, Thalia Otamendi, Noah D Silverberg, Esther Choi, Veronik Sicard, Roger Zemek, Katherine Healey, Olivier Brown, Lauren Butterfield, Andra Smith, Gary Goldfield, Rachel Kardish, Bechara Saab, Andrée-Anne Ledoux, Molly Cairncross

**Affiliations:** 1 Department of Psychology University of British Columbia Vancouver, BC Canada; 2 Department of Physical Therapy University of British Columbia Vancouver, BC Canada; 3 Children’s Hospital of Eastern Ontario Research Institute Ottawa, ON Canada; 4 Department of Pediatrics and Emergency Medicine University of Ottawa Ottawa, ON Canada; 5 School of Psychology Faculty of Social Sciences University of Ottawa Ottawa, BC Canada; 6 Department of Neuroscience Carleton University Ottawa, ON Canada; 7 Mobio Interactive Singapore Singapore; 8 Department of Cellular and Molecular Medicine University of Ottawa Ottawa, ON Canada; 9 Department of Psychology Simon Fraser University Burnaby, BC Canada

**Keywords:** adolescent, concussion, digital therapeutics, eHealth, mHealth, mindfulness, mobile health, social presence, working alliance

## Abstract

**Background:**

Despite the promising benefits of self-guided digital interventions for adolescents recovering from concussion, attrition rates for such interventions are high. Evidence suggests that adults can develop therapeutic alliance with self-guided digital interventions, which is in turn associated with intervention engagement. However, no research has examined whether adolescents develop therapeutic alliance with self-guided digital interventions and what factors are important to its development. Additionally, social presence—the extent to which digital encounters feel like they are occurring in person—may be another relevant factor to understanding the nature of the connection between adolescents and a self-guided digital intervention, though this has yet to be explored.

**Objective:**

This qualitative study explored the extent to which adolescents recovering from concussion developed therapeutic alliance and social presence during their use of a self-guided digital mindfulness-based intervention. Additionally, this study aimed to determine factors important to adolescents’ development of therapeutic alliance and social presence with the intervention.

**Methods:**

Adolescents aged between 12 and 17.99 years who sustained a concussion were recruited from 2 sites: a pediatric emergency department up to 48 hours after a concussion and a tertiary care clinic over 1 month following a concussion to capture adolescents who had both acute and persisting symptoms after concussion. Participants (N=10) completed a 4-week mindfulness-based intervention delivered through a smartphone app. Within the app, participants listened to audio recordings of mindfulness guides (voice actors) narrating psychoeducation and mindfulness practices. At 4 weeks, participants completed questionnaires and a semistructured interview exploring their experience of therapeutic alliance and social presence with the mindfulness guides in the intervention.

**Results:**

Themes identified within the qualitative results revealed that participants developed therapeutic alliance and social presence by “developing a genuine connection” with their mindfulness guides and “sensing real people.” Particularly important to the development of therapeutic alliance and social presence were the mindfulness guides’ “personal backgrounds and voices,” such that participants felt more connected to the guides by knowing information about them and through the guides’ calm tone of voice in audio recordings. Quantitative findings supported qualitative results; participants’ average score for therapeutic alliance was far above the scale midpoint, while the mixed results for social presence measures aligned with qualitative findings that participants felt that the mindfulness guides seemed real but not quite as real as an in-person connection would.

**Conclusions:**

Our data suggest that adolescents can develop therapeutic alliance and social presence when using digital interventions with no direct human contact. Adolescents’ development of therapeutic alliance and social presence with self-guided digital interventions can be bolstered by increasing human-like qualities (eg, real voices) within interventions. Maximizing therapeutic alliance and social presence may be a promising way to reduce attrition in self-guided digital interventions while providing accessible treatment.

## Introduction

Concussions are one of the most common injuries among adolescents, responsible for nearly 50,000 pediatric emergency department visits in Canada each year [1]. While most adolescents who sustain a concussion recover within 1 month, approximately 30% develop persistent postconcussion symptoms (PPCS) that continue longer than 1 month after their injury [2-4]. PPCS are associated with a variety of negative outcomes in adolescents, including reduced quality of life [5]. Despite the prevalence of PPCS and the detrimental effects that they have on the quality of life, few accessible evidence-based treatments exist for acute and persistent symptoms of concussion [6].

One promising intervention for those with acute or persisting concussion symptoms is mindfulness-based intervention (MBI) [7,8]. Most MBI research has focused on interventions delivered in-person. However, traditional in-person therapies are inaccessible for many due to financial barriers and sociodemographic disparities in access to health care. Moreover, a higher incidence of concussion in rural settings [9] means that many patients may have difficulty accessing specialized concussion care clinics, which are typically concentrated in urban settings [10]. Beyond geographical distance alone, specialized clinics often have long waitlists and operate solely during work and school hours, further complicating access to care. Additional barriers for adolescents to accessing treatment result from reliance on parental support for transport to and funding for in-person treatments. Finally, during infectious disease outbreaks such as the COVID-19 pandemic, in-person treatments may be severely limited or not available at all due to public health regulations. Taken together, in-person treatment for acute or persisting concussion symptoms in adolescents may not be readily accessible.

By contrast, self-guided digital interventions offer a potential solution to disparities in access to care for concussions in adolescents. A total of 95% of adolescents in Canada own a mobile digital device [11], highlighting that almost all youth in Canada have good access to digital care regardless of socioeconomic status. Digital interventions can be used anywhere and are a more affordable alternative to in-person treatments [12]. Some digital MBIs have also been shown to be effective in treating a range of conditions such as chronic pain, depression, anxiety, and stress [13,14], even when controlling for expectancy effects with active control comparison groups [15]. These conditions are also secondary consequences of concussion [2,16], which can further complicate the recovery process. Given digital MBIs’ effectiveness for treating these secondary consequences of concussion, they may have use for concussion recovery itself as well. However, attrition rates are high, and adherence suboptimal to digital interventions, with attrition rates as high as 52% in self-guided digital MBIs [12,17,18]. If patients do not adhere to treatment, they are less likely to benefit from it as intended. Thus, it is imperative to understand the factors underlying the large variation in self-guided MBI attrition rates.

One factor known to be essential for adherence and positive patient-reported outcomes following clinician-delivered behavioral interventions is the therapeutic alliance. Therapeutic alliance refers to the collaborative relationship and affective bond between a therapist and their patient [19,20]. The therapeutic alliance comprises 3 dimensions: the agreement on therapeutic goals, the agreement on therapeutic tasks, and the emotional bond between a therapist and their patient [21]. Particularly important to the conceptualization of therapeutic alliance is the collaborative nature of the relationship between a therapist and patient; this collaboration is integral to the success of psychotherapeutic treatments [19]. In face-to-face treatments, a strong therapeutic alliance is associated with positive treatment outcomes [20] and lower attrition [22].

Therapeutic alliance may also be relevant to patient adherence in the context of self-guided digital interventions. Self-guided digital interventions lack the direct contact with human therapists found in in-person treatments. However, research suggests that individuals often respond to digital technology as if it was another person [23]. As such, researchers have explored whether individuals may anthropomorphize—or attribute human-like qualities to—digital interventions themselves and therefore develop therapeutic alliance with the interventions. Indeed, initial evidence from interventions for adults focused on addressing a range of health behaviors and outcomes [24-31] suggests that individuals can develop therapeutic alliance when using self-guided digital interventions by feeling a connection to the intervention or digital social actors within it. Intervention format varied across studies; interventions included audio recordings of human voice actors [30], human-like digital avatars [25], fully automated text-based “conversations” with an intervention [28,29], and intervention content delivered through multimedia modules [24,26,27,31,32], highlighting that therapeutic alliance can develop with self-guided digital interventions in a variety of contexts. Additionally, evidence from quantitative studies of self-guided digital interventions indicates that therapeutic alliance is positively associated with patient engagement [27,32] and therapeutic outcomes [31,32]. This suggests that similar to in-person treatments, feeling socially supported is important to adherence in self-guided digital contexts as well. However, none of these studies sampled adolescents. Adolescents’ experiences of therapeutic alliance with self-guided digital interventions may differ from those of adults, given that adolescents engage with digital technology in unique ways from adults [33,34]. As such, whether and how adolescents develop therapeutic alliance when using self-guided digital interventions remains to be explored.

The nature of therapeutic alliance in self-guided digital interventions may differ from that developed during face-to-face treatments, given the lack of real-time interaction with a human therapist. For example, previous research found that while participants felt emotionally connected to and anthropomorphized their self-guided digital intervention, at other times, they felt that the intervention was a thing rather than a real person [29,30]. Holter’s relational model of “making come-alive” and “keeping un-alive” [28,29] can provide theoretical context as to why therapeutic alliance in self-guided interventions may be unique from therapeutic alliance developed in in-person therapy. The model suggests that individuals engage in 2 types of relational processes when using self-guided digital interventions: “making come-alive,” or thinking of a digital intervention as a social actor with human-like qualities, and “keeping un-alive,” or viewing the intervention as an inanimate object without real emotions. Individuals’ dynamic interplay between “making come-alive” and “keeping un-alive” produces a therapeutic alliance that at once both involves emotional closeness to a digital intervention and emotional distance through recognition that the program is not a real human entity.

Social presence may be a suitable construct for further understanding the therapeutic relationship and the extent to which individuals “make come-alive” or “keep un-alive” within self-guided digital interventions. Stemming from the communications and education literature, social presence is the extent to which a digital encounter feels like it is occurring in-person and has a sense of human connection to it [35-37]. Higher social presence is related to more trust in a web-based communicator [38] and greater relationship satisfaction, attachment, and perceived benefit of caregiving robots [39]. This suggests that social presence may approximate how much individuals experience a bond with social actors within a digital intervention. Social presence is also associated with satisfaction with, enjoyability of, and intention to use a mobile app or website [40-42]. Given the importance of these factors for adherence to treatment [43], attending to the social presence in self-guided digital intervention design may maximize adherence.

Social presence has not yet been examined in the context of a clinical intervention, and adolescents’ development of therapeutic alliance in the context of self-guided digital interventions is not well understood. This study sought to address these gaps in the literature and identify what factors may be relevant to adolescents’ development of therapeutic alliance and social presence in self-guided digital interventions. The aim of the present study was to determine if therapeutic alliance and social presence can be developed with audio-recorded mindfulness guides in a self-guided digital intervention among adolescents with acute or persisting concussion symptoms. Second, we aimed to determine what aspects of the intervention were important to the therapeutic alliance’s and social presence’s development.

## Methods

### Study Design

A qualitative exploratory design was used in the study. Since adolescents’ experiences of social presence and therapeutic alliance in clinical self-guided digital interventions are not yet well understood, we chose to use qualitative methods to explore the nuances of these experiences. Qualitative methods are well-suited for exploring participants’ experiences with digital applications, including what aspects of the application met their needs and what could improve their experience with the application. This was a planned secondary data analysis from an open-label pilot trial of a digital MBI for adolescents post concussion. The study was an open-label pilot trial because both the research team and participants knew that participants would be receiving a digital MBI. The pilot trial was a small-scale study and centered around the first stage of intervention development. The primary aim of the pilot trial was to gain information on the feasibility, credibility, and satisfaction of patient users with the digital MBI. The reporting of the study followed the Journal Article Reporting Standards for Qualitative Research (JARS-Qual) guideline [44].

### Research Team and Reflexivity

Interviews were conducted by KMKO, OB, and RK. KMKO conducted interviews with the PPCS cohort (adolescents experiencing PPCS) and OB and RK with the acute cohort (adolescents recovering from acute concussion). KMKO has a bachelor’s degree in psychology and was a directed studies student at the University of British Columbia at the time of the interviews. They were trained and supervised while conducting the interviews by MC, a postdoctoral fellow at the University of British Columbia at the time of data collection. MC holds a PhD in clinical psychology. OB has a bachelor’s degree in psychology and was a PhD candidate in experimental psychology at the time of the interviews. RK has a bachelor’s degree in neuroscience and mental health and was a master’s candidate at the time of the interview. They were trained and supervised while conducting the interviews by AAL, a scientist at the Children’s Hospital of Eastern Ontario Research Institute. AAL holds a PhD in experimental psychology.

Interviewers were specifically trained to conduct interviews in a nondirective manner and were given feedback from supervisors during practice interviews. Given the self-guided nature of the intervention, the research team remained open to the possibility that participants would not develop a therapeutic alliance or social presence with their mindfulness guides. If participants did not develop a therapeutic alliance or social presence when using the app, the research team planned to change the intervention during subsequent stages of intervention development based on participant feedback in this study to better support future users. As such, interviewers explored whether or not participants experienced therapeutic alliance and social presence with curiosity and openness to participant feedback. The participants and interviewers were unknown to each other before the study. At the beginning of the interviews, participants were made aware that the interviewers were research assistants affiliated with the research team that conducted the study. They were told that the researcher was interested in learning about their experiences while using the mindfulness app.

Qualitative data were analyzed by KMKO and TO. At the time of data analysis, KMKO had approximately 2 years of experience conducting research about patient experiences with digital interventions. Based on their professional background and familiarity with the therapeutic alliance and social presence literature, KMKO brought certain assumptions to data analysis. For example, KMKO expected that participants would likely develop some degree of therapeutic alliance and social presence with the mindfulness guides given the human-like qualities present in audio recordings of the guides (eg, using real human voices). Additionally, because KMKO conducted interviews with the PPCS cohort, they had familiarity with the experiences of the PPCS cohort before beginning data analysis. Due to KMKO’s awareness of their preconceived expectations, they exercised caution during data analysis and engaged in constant peer debriefing with TO to ensure that findings were indeed based on participants’ accounts. TO is a Rehabilitation Sciences PhD student at the University of British Columbia who is trained in qualitative methodologies and has a background in psychology. TO was not versed in the therapeutic alliance, social presence, or digital intervention literature and did not conduct any of the interviews. As such, TO had limited assumptions about the findings going into data analysis.

### Participants

The sample included 10 adolescents aged between 12 and 17.99 years who were either recovering from acute concussion (acute cohort) or were experiencing PPCS (PPCS cohort). The sample included both the acute cohort and the PPCS cohort because the digital MBI was designed both to help adolescents acutely recovering from concussion and those with persisting symptoms. As such, one of the primary aims of the pilot trial was to understand whether the digital intervention met the needs of both populations. In this study, we did not expect the acute cohort and PPCS cohort to differ with respect to their experiences of therapeutic alliance and social presence. Therefore, the 2 cohorts were collapsed into 1 overall sample for data analysis.

The acute and PPCS cohorts were recruited from 2 separate sites. Participants in the acute cohort (n=7) were recruited from the emergency department within 48 hours of sustaining a concussion. Participants in the PPCS cohort (n=3) were youth who sustained a concussion between 1 and 12 months before participation in the study and continued to experience concussion symptoms in at least 3 categories, as defined by the *International Classification of Diseases, Tenth Revision* criteria for PPCS [45]. They were recruited from a tertiary care concussion clinic. Additional eligibility criteria for the study included that participants were proficient in English and had access to a smartphone or tablet with an internet connection. Participants were excluded if they had a severe chronic neurological developmental delay resulting in communication difficulties, previous psychiatric hospitalization, previous diagnosis of a severe psychiatric disorder, such as schizophrenia (acute cohort only), or if participants were currently involved in other MBIs (PPCS cohort only). The acute and PPCS cohorts differed in size due to challenges associated with recruiting participants experiencing chronic concussion symptoms. In particular, given that recruitment was carried out during the COVID-19 pandemic, anecdotally, adolescents with PPCS faced greater barriers to care than usual, such as increased family stress and restricted access to the tertiary care concussion clinic where recruitment was carried out due to public health regulations.

Participants’ demographic information is summarized in Table 1. A total of 10 adolescents (mean age 15.93, SD 1.86 years) participated in our study. Overall, 7 participants identified as female, and 3 participants identified as male. Participants are described using pseudonyms to protect their identities.

**Table 1:** Participant demographic characteristics of adolescents recovering from concussion. Participant names presented in this table are pseudonyms to protect the participants’ anonymity.

Cohort and participants		Sex	Age (years)
**Acute**			
	Sophia	Female	17.22
	Julian	Male	16.62
	June	Female	13.94
	Ella	Female	14.26
	Olivier	Male	16.94
	Marie-Claude	Female	12.63
	Gabrielle	Female	14.73
**Persistent postconcussion symptoms**			
	Alex	Male	17.86
	Ada	Female	17.48
	Kay	Female	17.62

### Ethical Considerations

This study’s procedures were reviewed and granted ethics approval by the Behavioral Research Ethics Board at the University of British Columbia (H20-00120) and the Research Ethics Board at the Children’s Hospital of Eastern Ontario Research Institute (20/72X). Participants capable of consenting provided their informed consent, and parents also provided consent on their children’s behalf. Participants who were unable to consent provided assent instead, and their parents provided informed consent on their behalf. Participants were able to ask the research assistant questions that they had about the study procedures before providing their consent. With respect to study data, all participants were assigned a unique participant ID number that was linked to their data; as such, all study data were deidentified and stored in the secure platform, Research Electronic Data Capture (REDCap) [46,47]. Participants in the acute cohort were compensated for their participation with a CAD $10 (US $7.40) gift card and received a letter stating that they completed 10 hours of volunteer work, while those in the PPCS cohort were given a CAD $15 (US $11.11) gift card. Compensation differed between the 2 cohorts because they were recruited from separate sites, with data collection for each cohort led by teams at 2 different institutions. As such, the cohorts were compensated in accordance with the norms of the given institution leading each cohort’s data collection.

### Procedure

Prospective participants for the acute cohort were approached in the emergency department. A member of the research team briefly described the study. Interested prospective participants were called by telephone by a research assistant for the eligibility screening and consent or assent process the next day.

Prospective participants for the PPCS cohort were approached at web-based psychoeducation sessions at a tertiary care concussion clinic. A research assistant provided prospective participants with information about the study and gave all participants a link to a survey on the secure platform, REDCap. The survey allowed patients to provide consent to be contacted by the research team if they were interested in participating in the study. Interested prospective participants were called by telephone by a research assistant for the eligibility screening and consent or assent process.

Upon confirming participants’ eligibility and receiving informed consent, participants were emailed a unique link to register and download the intervention through REDCap. The intervention was developed by the research team and is hosted on the AmDTx app. The app was downloaded on participants’ smartphone or tablet, along with instructions for use. A research assistant also called participants by telephone to help instruct them through the app download process and answer any technical questions they had. Concurrently, participants were emailed links to complete baseline measures through REDCap. All data were collected and stored in a secure database on REDCap.

Participants then engaged with the intervention at their own pace over the course of 4 weeks, following which they received a link to complete outcome measures assessing social presence and therapeutic alliance on REDCap. After participants finished the outcome measures, they completed a 30-45–minute semistructured interview through Zoom (Zoom Video Communication) video call with a member of the research team. Audio of the interviews was recorded for transcription.

### Intervention

During this pilot phase, participants completed 4 weeks of the 8-week treatment. The recordings of psychoeducation and mindfulness practices in the app were narrated by 2 professional mindfulness guides, Ruby and Brian. Ruby and Brian both have substantial mindfulness training and teaching experience. Moreover, Ruby (who led all the psychoeducation) had experience working with adolescents with concussion. The MBI was modeled based on mindfulness-based stress reduction [48] with meditations and content specifically designed for adolescents with concussion. The psychoeducation topics covered in the MBI involved pain acceptance, stress management, and nonjudgement toward concussion symptoms. Mindfulness practices included mindful breathing, body scans, and mindful movement. Participants listened to the audio recordings of the mindfulness guides narrating the psychoeducation sessions and mindfulness exercises while using the app. Participants were encouraged to participate in the intervention for 15 minutes per day, for 4 days a week. The content was the same for all participants. See Multimedia Appendix 1 for images of the intervention content.

Throughout treatment, participants also received weekly SMS text messaging support from an “SMS text messaging coach,” who was a trained member of the research team. The SMS text messaging coach’s role was to support participants with any technical issues and problem-solve any barriers to engagement, such as having difficulty finding time to complete their mindfulness practices. SMS text messages sent to participants followed a highly templated SMS text messaging protocol. For a copy of the SMS text messaging protocol, see Multimedia Appendix 2.

### Measures

#### Therapeutic Alliance

Therapeutic alliance was measured using the Working Alliance Inventory adapted for Guided Internet Interventions (WAI-I). The WAI-I is an adaptation of the Working Alliance Inventory–Short Form Revised designed for use with digital interventions. It is a self-reported, 12-item measure assessing participants’ alignment on tasks and goals with a digital intervention and their bond with their mindfulness guides during the intervention [49]. In this study, items were modified to reflect the MBI at hand (eg, “The goals of the app-based mindfulness program are in line with my goals”), including replacing the word “psychologist” in the original measure with “mindfulness guides” (eg, “The mindfulness guides who support me in the app-based program are really interested in my well‐being”). Each item was assessed using a 5-point Likert scale ranging from 1 (never) to 5 (always). WAI-I total scores were calculated by summing items out of a possible 60 points. The WAI-I is a reliable measure with good construct, convergent, discriminant, and external validity [49].

#### Social Presence

In order to evaluate distinct aspects of social presence, 2 measures were used to capture how much digital encounters feel like they are occurring in-person and the ways in which digital encounters have human-like qualities. The first measure was adapted from Nowak and Biocca’s self-reported, 6-item Perceived Social Presence (PSP) scale [36]. The measure assessed how much participants felt like their mindfulness guides were real people and how much it felt like they were “with” their guides while using the app (ie, “To what extent was this as if you were in the same room with your mindfulness guide?”) [36]. We modified the wording of items to reflect that participants were reporting about their experiences with the mindfulness guides, as opposed to the wording “partner” used in the original measure. Items were measured on a Likert scale ranging from 1 (no extent) to 7 (full extent). The measure was scored by summing items out of a possible 42 points. The PSP scale has good internal consistency, convergent validity, and discriminant validity [36,50].

The second measure was the self-reported, 17-item Gunawardena Social Presence Indicators (SPI) scale [51] which assessed the human-like qualities of the audio-recorded mindfulness guides in the intervention. Participants were presented with 5-point bipolar items consisting of contrasting adjectives (ie, “personal-impersonal”) [51] and were instructed to circle the number that best reflected their perceptions of their mindfulness guides’ interpersonal qualities for each item. The measure was scored by summing items out of a possible 85 points. Higher scores reflected more negative perceptions of mindfulness guides. The SPI scale demonstrates good convergent validity [52,53].

#### Semistructured Interview

A semistructured, qualitative interview was developed by the research team to better understand participants’ experience with the app. Before the study’s commencement, the interview was pilot-tested with a 15-year-old nonparticipant to obtain feedback about understandability and was also reviewed by expert clinicians and researchers for content. The interview covered topics such as participants’ experience of therapeutic alliance and social presence with the mindfulness guides while using the app, as well as changes they would make to the app to improve those experiences (ie, “Can you tell me about your connection with your mindfulness guides? Is there anything you would have liked to change about your connection with your mindfulness guides?”). Other topics explored in the interview included participants’ satisfaction with the app content and ease of use and their satisfaction with support received from their SMS text messaging coaches, though data gathered from these questions is outside the scope of this study (for more information, see [54]). For a copy of the interview guide, see Multimedia Appendix 3.

### Data Analysis

#### Quantitative Analysis

Participant demographics were summarized using descriptive and frequency statistics, as appropriate. Scores on the WAI-I and the PSP scale represent the degree to which participants developed therapeutic alliance and experienced social presence when using the app. As such, scores above the scales’ floor suggest participants developed some degree of therapeutic alliance and social presence. The scale midpoint was used for the SPI scale to indicate whether participants viewed their mindfulness guides more positively, neutrally, or negatively. We chose this method of examining our quantitative results because traditional quantitative data analysis was not appropriate for our data, given our small sample size. The quantitative results are used to supplement the main, qualitative results stemming from the semistructured interview by demonstrating the degree to which participants did or did not develop therapeutic alliance and social presence when using the intervention.

#### Qualitative Analysis

Qualitative interviews were audio recorded and transcribed verbatim using NVivo 12 software (Lumivero). Following transcription, interviews were analyzed by KMKO and TO using conventional content analysis [55]. The researchers first read each interview multiple times to familiarize themselves with the data. Transcripts were then open-coded, codes were grouped into meaningful clusters, and these clusters were used to form overarching categories that related to social presence and therapeutic alliance. Categories were discussed, refined, and finalized in consultation with MC. Content analysis allows for a mix of deductive and inductive analysis [56]. Deductive analysis was used to code transcripts based on understandings of social presence and therapeutic alliance gained from previous literature, while inductive analysis was used to explore how social presence and therapeutic alliance developed and were important in the novel context of self-guided digital interventions. Representative quotes were chosen to supplement the written findings.

## Results

### Quantitative Results

A total of 9 (90%) participants completed all quantitative measures. Descriptive values for the quantitative questionnaires are presented in Table 2. Participants’ mean therapeutic alliance score on the WAI-I was far above the WAI-I scale floor, suggesting that in general, our participants did develop therapeutic alliance with the mindfulness guides. The mean score for the bond subscale of the WAI-I was particularly high, suggesting that participants especially developed a sense of bonding with their guides, relative to their sense of agreement with guides on tasks or goals in the intervention.

Participants’ mean social presence score on the PSP scale was far above the scale floor, suggesting that participants developed a social presence with their mindfulness guides. Participants’ average score on the SPI scale was slightly above its scale midpoint—with lower scores indicating greater social presence. This result indicates that participants had fairly neutral impressions of their mindfulness guides’ interpersonal characteristics and the social presence experienced with the guides.

**Table 2:** Descriptive values for questionnaire outcomes from adolescents recovering from concussion using a digital mindfulness-based intervention.

Measure		Minimum-Maximum	Mean (SD)	Scale midpoint	Scale range
**WAI-I^a^**					
	Total score	38.00-59.00	47.00 (6.80)	24.00	12.00-60.00
	Task and goal agreement	20.00-39.00	30.33 (6.34)	16.00	8.00-40.00
	Bonding with guides	12.00-20.00	16.67 (2.55)	8.00	4.00-20.00
**PSP^b^**					
	Total score	11.00-39.00	24.40 (7.55)	17.50	6.00-30.00
**SPI^c^**					
	Total score^d^	19.00-58.00	35.89 (10.63)	34.00	17.00-85.00

^a^WAI-I: Working Alliance Inventory for Guided Internet Interventions.

^b^PSP: Perceived Social Presence scale.

^c^SPI: Social Presence Indicators Scale.

^d^A lower score indicates a more positive social presence.

### Qualitative Results

#### Developing a “Genuine Connection”

When asked about their mindfulness guides, many participants readily described developing an important emotional bond with their guides. For example, Gabrielle said that their connection with the guides “didn’t feel artificial... it felt like it was a genuine connection.” Olivier expressed, “I like that they kind of got what the listener was feeling... they had pretty good insight into various different things that... I was feeling.” Sophia also indicated feeling a sense of safety with their guides, stating, “I don’t know how to describe it. [The connection is] like feeling safe with someone.” This was echoed by Ella, who stated that listening to the guides “was just kind of like a comfortable situation, and you didn’t really feel like there was anything wrong.”

The participants further elaborated on how this connection with the guides helped them progress through the mindfulness program. Alex noted, “It just makes you feel like they were trying to help you the whole time. Like they want you to accomplish your goals... you can definitely tell that they’re like guiding you through.” Olivier highlighted that the guides also normalized challenges that arose during the program, citing 1 example where the guides said, “If you’re thinking that you’re bad at mindfulness, it’s normal for your thoughts to wander and all that. [The guides] just kind of understood.” This experience was shared with Kay, who said, “I would lose track of what I was doing, and they’re like, if you lose track like it’s okay, just come back to what we’re doing, think about this.” And I’m like, “Okay, thank you.” The use of certain adjectives when describing the guides, such as “understanding” (Ella, Julian, and Olivier), “caring” (Ada and Olivier), “helpful” (Alex and Sophia), and “friendly” (Alex and Kay), further exemplified the participants’ feeling of being taken care of by the guides, which facilitated their progression through the program.

#### Sensing “Real People”

The use of human-like adjectives to describe the mindfulness guides indicates that participants viewed the guides as real people. Indeed, participants elaborated on the perceived realness of the guides. For example, Gabrielle mentioned that Ruby “felt like... a real person, like really genuine.” Ada also contrasted the realness of the guides to the absence of realness in automated computer voices; they said, “This is a person and like, again, not an automated voice message... it was at one point another person on the other side of the screen, it’s not like Siri talking back to you.” One participant even stated, “I would be surprised if [you] told me they weren’t real people” (Kay). Some participants additionally assigned a particular identity role to their guides, saying that their guides felt like “teachers” (Olivier), or “a parental figure” (Gabrielle); beyond feeling like real people, the mindfulness guides also felt like particular kinds of people for some participants.

In addition to feeling like their guides were real people, participants also noted that their experiences with the guides felt like real-life interactions. For example, Ada offered that it “felt like someone was talking in my ear when I was like, lying down and things... it definitely felt like there was like somebody there.” Alex highlighted that “it feels like they’re actually talking to you.” Gabrielle said “It felt like they were like right in front of me, too. So like, presence.” Multiple participants noted that the interactions with the guides felt private and personal, with both Alex and Ella describing that their mindfulness sessions with the guides felt like a “one-on-one talk” and Olivier saying that sessions “just felt like more like a conversation that they’re having with me, and it just felt like as though they’re talking to me rather than just talking and letting someone hear.”

As participants elaborated on their guides, however, tensions in their accounts were palpable when describing their ability to develop feelings of presence and connection despite the guides not actually being present. Ella offered, “This is probably going to sound weird, but [they were] understanding even though you couldn’t talk to them. It kind of felt like they were listening.” When asked to what extent it felt like the guides were present during the intervention, Sophia answered, “I feel like they’re with me, but not with me at the same time, because I know they’re not with me.” Ada noted that contextual factors were relevant to whether they felt like their guides were present or not, stating, “I don’t think I felt [like the guides were talking in my ear] when I was walking, because when I’m walking, I’m like, nine times out of ten having to pay attention to my areas around me... But when I was lying down, it was definitely there.” Kay also stated, “I wasn’t connected, but I wasn’t not connected.” The dissonance in the participants’ narrative accounts alluded to the sense that participants could not help but feel a connection with their guides but also did not want to deceive themselves into thinking that these connections were real—or at least, quite as real as an in-person connection would be. Indeed, Sophia said that “it felt like I was on a phone call with someone,” suggesting that while their interactions with guides did not necessarily feel like they were happening in-person, their relationship with the guides still felt more interactive than the completely noninteractive connection they actually had.

#### Personal Backgrounds and Voices

The mindfulness guides’ backgrounds and tone of voice emerged as factors key to the development of participants’ bond and sense of presence with the guides.

Getting to know the guides allowed the participants to “trust them” (Olivier) by getting the sense that the guides “knew what they were talking about” (Olivier). Some participants trusted Brian due to his personal experience with concussion. As Alex noted, Brian’s experience of having concussions in the past made him seem very “personable,” as participants felt that it was not “just someone like talking to you about it, ’it’s someone who’s actually experienced [concussion]” (Alex). Others trusted Ruby because of her professional and educational background, for example, because she mentioned her degree in psychology. Ada noted, “Obviously [Ruby] knew about concussions. And I respected that.” The sharing of the personal or professional backgrounds of the guides allowed participants to feel like the guides had “either been through this (ie, a concussion experience) or they’ve worked with other people who’ve been through this,” (Kay) which allowed them to trust the trajectory and goals of the intervention.

Most participants also expressed that the guides’ voices were important for helping them get to know the guides and making them feel real. Ada captured this sentiment by sharing, “I definitely think that you just kind of get to know a person by the way that they speak.” The most common descriptors of the guides’ voices were “calm” or “calming” (Sophia, Ella, Olivier, Marie-Claude, Sophia, Ada, and Kay) and “soothing” (Julian, Olivier, and Ada). When probed about why this tone of voice was important, participants shared that a calming voice allowed them to feel like they were being cared for, and this had important implications for the development of trust in the guides. For example, Sophia elaborated, “I think them being calm with their voices made me closer to feel like they’re actually here to help me instead of being rushed and stuff.” Thus, participants highlighted that factors that allowed them to get to know their mindfulness guides (ie, personal backgrounds and tone of voice) were beneficial for the development of their therapeutic alliance and sense of social presence, which in turn had benefits for their engagement with the app.

## Discussion

### Overview

This study aimed to determine whether and how therapeutic alliance and social presence could develop in the context of a self-guided digital MBI for adolescents post concussion. Qualitative and quantitative findings revealed that adolescents did develop therapeutic alliance through their use of the intervention, with qualitative findings providing insight into factors important for its development; participants readily described experiencing a bond with the mindfulness guides in the intervention. While participants’ average score on the SPI scale was slightly above the scale midpoint, this may be explained by nuances uncovered with qualitative findings, which highlighted the tension in how the participants experienced social presence with their guides (eg, it felt real, but not quite as real as in-person connections). Indeed, this tension aligns with Holter’s relational model [29], suggesting that individuals’ connections with their mindfulness guides in this digital context involved both “making come-alive” and “keeping un-alive.” This, along with participants’ high average score on the PSP scale, suggests that participants did develop social presence during their use of the intervention. The guides’ personal backgrounds and the tone of their voices emerged as particularly important factors in the development of therapeutic alliance and social presence in a digital context. Additionally, participants stated that their connection with the guides and sense of the guides’ realness helped maintain their engagement with and interest in the intervention. Results extend previous findings that adults can develop therapeutic alliance with self-guided digital interventions [24-31] by demonstrating that adolescents also develop therapeutic alliance in self-guided digital contexts. Overall, findings highlighted the potential feasibility and use of maximizing therapeutic alliance and social presence to bolster participant engagement with self-guided digital interventions.

Since the onset of the COVID-19 pandemic, clinicians have identified the need for accessible, engaging, and effective digital interventions [57,58]. Digital interventions circumvent a number of accessibility barriers that limit adolescents’ access to concussion care. Therefore, they represent a promising treatment option for both acute and persistent concussion symptoms. However, numerous studies have noted particularly high attrition rates for self-guided digital interventions, which researchers have suggested are because of their limited human contact and lack of social support [12,17,18]. Our findings demonstrate that even in self-guided digital interventions, participants can develop therapeutic alliance and a sense of social presence in the program with audio recordings of mindfulness guides. This suggests that, despite the absence of a human for participants to directly interact with, adolescents can still experience being socially supported through self-guided interventions. Thus, it is important to consider ways of maximizing the human support participants feel during self-guided digital interventions to bolster participant engagement.

In self-guided contexts, factors that anthropomorphize digital guides—in other words, make them feel more human—emerged as particularly important to the development of therapeutic alliance and social presence among our sample. Participants highlighted that learning about the guides’ backgrounds (either their experiences of concussions or their background in psychology) helped them to feel connected to guides and trust that the content of the intervention would help them work toward their goals—both key aspects of therapeutic alliance. The guides’ voices also emerged as a salient factor that made guides feel like real people to participants, demonstrating that the “realness” of guides’ voices was key to the development of social presence. Indeed, when asked whether guides’ voices sounded like those of real people, every participant except 1 (Sophia) said yes. Thus, factors that increased participants’ ability to anthropomorphize their guides stood out as a potential mechanism important to the development of both therapeutic alliance and social presence in self-guided digital interventions. Our data align with quantitative findings that individuals experience greater social presence when digital conversational agents are characterized by higher levels of anthropomorphic cues (eg, human-like voices) [59]. Potentially, a higher level of anthropomorphic cues in self-guided digital interventions better enables individuals to “make come-alive” [28,29].

### Recommendations

Our findings have several implications for researchers developing self-guided digital interventions for mental health concerns. Given that anthropomorphizing guides emerged as particularly important to the development of both therapeutic alliance and social presence in our sample, we recommend multiple strategies to increase the human-like qualities of audio-recorded guides in self-guided digital interventions. First, providing information about the guides’ personal connection with the topic of focus of the intervention (eg, concussion knowledge in this study) will promote how relatable and trustworthy guides seem to participants. Our findings suggest that guides either having personal experience with the topic of the intervention or having a background in psychology (and thus, seeming knowledgeable to participants about the intervention) may both be promising ways for mindfulness guides to encourage connection with participants and seem more real. Second, using real human actors to record audio for guides’ voices, as was done in our intervention, will seem more realistic and promote a greater bond with guides compared to using computerized voices. Guides may benefit from training in clinical microskills (eg, conveying warmth, genuineness, and empathy) [60] to ensure that their tone of voice is calming and soothing, both of which were identified by our participants as important attributes in guides’ voices for therapeutic alliance and social presence. Additionally, guides can facilitate participants’ sense of connection with them by anticipating and addressing potential challenges that may arise for participants. Participants in our sample reported that this helped them feel understood by the guides. Lastly, 1 key addition participants in our study suggested to increase their sense of connection with and humanness of guides was to show pictures of the guides’ faces. Indeed, previous research suggests that elements of a digital interface’s design—for example, providing identity cues like names and photos of the communicators within the interface—increase social presence [38,61]. Therefore, having an image of the voice actors who are the mindfulness guides may better allow participants to develop a narrative of who their guides are, impacting both the connection they feel to their guides (therapeutic alliance) and the extent to which the guides feel like real people (social presence).

### Limitations and Future Directions

Given our small sample size, our findings are limited with respect to the quantitative analyses we were able to conduct. While it was a secondary focus of our study, having a larger sample may have allowed us to conduct formal quantitative analyses as opposed to the descriptive analyses presented in this paper. Future research can use quantitative methods with a larger sample to formally explore the independent and interactive roles that therapeutic alliance and social presence play in the development of self-guided digital interventions.

Future research may also benefit from building upon this study’s results and previous research on adults [28] by exploring whether individual differences impact the extent to which adolescents experience therapeutic alliance and social presence during self-guided digital interventions and whether they benefit from experiencing them. Adolescents may differ in how much they desire and benefit from human support in digital interventions. For example, therapeutic alliance and social presence may be less important to the engagement of participants who are more introverted and potentially desire human support in therapeutic contexts to a lesser extent. Research can also examine whether sharing characteristics with guides (eg, race or ethnicity and gender) or not impacts how much participants develop therapeutic alliance and social presence with their guides. Potentially, participants may relate to guides who share their characteristics more, thus developing more of a connection with them.

### Conclusions

This study found that adolescents with concussion develop therapeutic alliance and social presence in self-guided digital interventions and identified factors key to their development (eg, tone of voice and personal backgrounds). Our study is one of the first to examine the role of social presence in a clinical context with adolescents and demonstrate the use of social presence for encouraging adolescent engagement with self-guided digital interventions. Future research should focus on maximizing therapeutic alliance and social presence for digital intervention development by increasing the number of human-like qualities that guide the intervention process. This may address key concerns about self-guided digital interventions—high attrition rates and low participant engagement—to maximize their effectiveness. Doing so will allow for the development of digital interventions that are more accessible and less resource-intensive than in-person treatments, as well as being engaging for users. Ultimately, understanding the development of therapeutic alliance and social presence within self-guided digital interventions aids in the development of accessible and engaging digital treatments—one digital connection at a time.

## Appendix

Multimedia Appendix 1 Images of intervention content.

Multimedia Appendix 2 Texting protocol.

Multimedia Appendix 3 Semistructured interview.

## Abbreviations

JARS-Qual: Journal Article Reporting Standards for Qualitative Research
MBI: mindfulness-based intervention
PPCS: persistent postconcussion symptoms
PSP: Perceived Social Presence
REDCap: Research Electronic Data Capture
SPI: Social Presence Indicators
WAI-I: Working Alliance Inventory adapted for Guided Internet Interventions

## Data Availability

The data analyzed during this study are not publicly available in order to protect the privacy and confidentiality of participants, given the small sample size. However, data are available from the corresponding author on reasonable request.

## References

[1] (2020). Concussion: sport and recreation. Public Health Agency of Canada.

[2] Babcock L, Byczkowski T, Wade SL, Ho M, Mookerjee S, Bazarian JJ (2013). Predicting postconcussion syndrome after mild traumatic brain injury in children and adolescents who present to the emergency department. JAMA Pediatr.

[3] Ledoux AA, Tang K, Yeates KO, Pusic MV, Boutis K, Craig WR, Gravel J, Freedman SB, Gagnon I, Gioia GA, Osmond MH, Zemek RL (2019). Natural progression of symptom change and recovery from concussion in a pediatric population. JAMA Pediatr.

[4] Zemek R, Barrowman N, Freedman SB, Gravel J, Gagnon I, McGahern C, Aglipay M, Sangha G, Boutis K, Beer D, Craig W, Burns E, Farion KJ, Mikrogianakis A, Barlow K, Dubrovsky AS, Meeuwisse W, Gioia G, Meehan WP, Beauchamp MH, Kamil Y, Grool AM, Hoshizaki B, Anderson P, Brooks BL, Yeates KO, Vassilyadi M, Klassen T, Keightley M, Richer L, DeMatteo C, Osmond MH (2016). Clinical risk score for persistent postconcussion symptoms among children with acute concussion in the ED. JAMA.

[5] Novak Z, Aglipay M, Barrowman N, Yeates KO, Beauchamp MH, Gravel J, Freedman SB, Gagnon I, Gioia G, Boutis K, Burns E, Ledoux AA, Osmond MH, Zemek RL (2016). Association of persistent postconcussion symptoms with pediatric quality of life. JAMA Pediatr.

[6] Polinder S, Cnossen MC, Real RGL, Covic A, Gorbunova A, Voormolen DC, Master CL, Haagsma JA, Diaz-Arrastia R, von Steinbuechel N (2018). A multidimensional approach to post-concussion symptoms in mild traumatic brain injury. Front Neurol.

[7] Acabchuk RL, Brisson JM, Park CL, Babbott-Bryan N, Parmelee OA, Johnson BT (2021). Therapeutic effects of meditation, yoga, and mindfulness-based interventions for chronic symptoms of mild traumatic brain injury: a systematic review and meta-analysis. Appl Psychol Health Well Being.

[8] Paniccia M, Knafo R, Thomas S, Taha T, Ladha A, Thompson L, Reed N (2019). Mindfulness-based yoga for youth with persistent concussion: a pilot study. Am J Occup Ther.

[9] Yue JK, Upadhyayula PS, Avalos LN, Phelps RRL, Suen CG, Cage TA (2020). Concussion and mild-traumatic brain injury in rural settings: epidemiology and specific health care considerations. J Neurosci Rural Pract.

[10] Ellis MJ, Russell K (2019). The potential of telemedicine to improve pediatric concussion care in rural and remote communities in Canada. Front Neurol.

[11] Table 22-10-0115-01 smartphone use and smartphone habits by gender and age group, inactive. Statistics Canada.

[12] Mrazek AJ, Mrazek MD, Cherolini CM, Cloughesy JN, Cynman DJ, Gougis LJ, Landry AP, Reese JV, Schooler JW (2019). The future of mindfulness training is digital, and the future is now. Curr Opin Psychol.

[13] Jayewardene WP, Lohrmann DK, Erbe RG, Torabi MR (2017). Effects of preventive online mindfulness interventions on stress and mindfulness: a meta-analysis of randomized controlled trials. Prev Med Rep.

[14] Russell L, Ugalde A, Milne D, Austin D, Livingston PM (2018). Digital characteristics and dissemination indicators to optimize delivery of internet-supported mindfulness-based interventions for people with a chronic condition: systematic review. JMIR Ment Health.

[15] Walsh KM, Saab BJ, Farb NA (2019). Effects of a mindfulness meditation app on subjective well-being: active randomized controlled trial and experience sampling study. JMIR Ment Health.

[16] Ayr LK, Yeates KO, Taylor HG, Browne M (2009). Dimensions of postconcussive symptoms in children with mild traumatic brain injuries. J Int Neuropsychol Soc.

[17] Fish J, Brimson J, Lynch S (2016). Mindfulness interventions delivered by technology without facilitator involvement: what research exists and what are the clinical outcomes?. Mindfulness (N Y).

[18] Linardon J, Fuller-Tyszkiewicz M (2020). Attrition and adherence in smartphone-delivered interventions for mental health problems: a systematic and meta-analytic review. J Consult Clin Psychol.

[19] Horvath AO, Del Re AC, Flückiger C, Symonds D (2011). Alliance in individual psychotherapy. Psychotherapy (Chic).

[20] Martin DJ, Garske JP, Davis MK (2000). Relation of the therapeutic alliance with outcome and other variables: a meta-analytic review. J Consult Clin Psychol.

[21] Bordin ES The working alliance: basis for a general theory of psychotherapy.

[22] Sharf J, Primavera LH, Diener MJ (2010). Dropout and therapeutic alliance: a meta-analysis of adult individual psychotherapy. Psychotherapy (Chic).

[23] Reeves B, Nass C (1996). The Media Equation: How People Treat Computers, Television, and New Media Like Real People and Places.

[24] Barazzone N, Cavanagh K, Richards DA (2012). Computerized cognitive behavioural therapy and the therapeutic alliance: a qualitative enquiry. Br J Clin Psychol.

[25] Bickmore T, Gruber A, Picard R (2005). Establishing the computer-patient working alliance in automated health behavior change interventions. Patient Educ Couns.

[26] Brandt CL, Dalum P, Thomsen TT (2013). "I miss the care even though I know it's just a machine": an explorative study of the relationship between an internet-based smoking cessation intervention and its participants. Health Informatics J.

[27] Clarke J, Proudfoot J, Whitton A, Birch MR, Boyd M, Parker G, Manicavasagar V, Hadzi-Pavlovic D, Fogarty A (2016). Therapeutic alliance with a fully automated mobile phone and web-based intervention: secondary analysis of a randomized controlled trial. JMIR Ment Health.

[28] Holter MTS, Ness O, Johansen A, Brendryen H (2019). Getting change-space: a grounded theory study of automated eHealth therapy. Qual Rep.

[29] Holter MTS, Ness O, Johansen AB, Brendryen H (2020). Making come-alive and keeping un-alive: how people relate to self-guided web-based health interventions. Qual Health Res.

[30] Kaplan B, Farzanfar R, Friedman RH (2003). Personal relationships with an intelligent interactive telephone health behavior advisor system: a multimethod study using surveys and ethnographic interviews. Int J Med Inform.

[31] Meyer B, Bierbrodt J, Schröder J, Berger T, Beevers CG, Weiss M, Jacob G, Späth C, Andersson G, Lutz W, Hautzinger M, Löwe B, Rose M, Hohagen F, Caspar F, Greiner W, Moritz S, Klein JP (2015). Effects of an internet intervention (deprexis) on severe depression symptoms: randomized controlled trial. Internet Interv.

[32] Cavanagh K, Belnap BH, Rothenberger SD, Abebe KZ, Rollman BL (2018). My care manager, my computer therapy and me: the relationship triangle in computerized cognitive behavioural therapy. Internet Interv.

[33] Fernández-Ardèvol M, Belotti F, Ieracitano F, Mulargia S, Rosales A, Comunello F (2020). “I do it my way”: idioms of practice and digital media ideologies of adolescents and older adults. New Media Soc.

[34] Moreno MA, Binger K, Zhao Q, Eickhoff J, Minich M, Uhls YT (2022). Digital technology and media use by adolescents: latent class analysis. JMIR Pediatr Parent.

[35] Biocca F, Harms C, Burgoon JK (2003). Toward a more robust theory and measure of social presence: review and suggested criteria. Presence.

[36] Nowak KL, Biocca F (2003). The effect of the agency and anthropomorphism on users' sense of telepresence, copresence, and social presence in virtual environments. Presence.

[37] Short J, Williams E, Christie B (1976). The Social Psychology of Telecommunications.

[38] Hess TJ, Fuller M, Campbell DE (2009). Designing interfaces with social presence: using vividness and extraversion to create social recommendation agents. J Assoc Inf Syst.

[39] Kim KJ, Park E, Sundar SS (2013). Caregiving role in human–robot interaction: a study of the mediating effects of perceived benefit and social presence. Comput Human Behav.

[40] Hassanein K, Head M (2007). Manipulating perceived social presence through the web interface and its impact on attitude towards online shopping. Int J Hum Comput Stud.

[41] Lazard AJ, Brennen JS, Adams ET, Love B (2020). Cues for increasing social presence for mobile health app adoption. J Health Commun.

[42] Park S, Cho K, Lee BG (2014). What makes smartphone users satisfied with the mobile instant messenger?: Social presence, flow, and self-disclosure. Int J Multimed Ubiquitous Eng.

[43] Barbosa CD, Balp MM, Kulich K, Germain N, Rofail D (2012). A literature review to explore the link between treatment satisfaction and adherence, compliance, and persistence. Patient Prefer Adherence.

[44] Levitt HM, Bamberg M, Creswell JW, Frost DM, Josselson R, Suárez-Orozco C (2018). Journal article reporting standards for qualitative primary, qualitative meta-analytic, and mixed methods research in psychology: the APA Publications and Communications Board task force report. Am Psychol.

[45] (1993). The ICD-10 Classification of Mental and Behavioural Disorders: Diagnostic Criteria for Research.

[46] Harris PA, Taylor R, Minor BL, Elliott V, Fernandez M, O'Neal L, McLeod L, Delacqua G, Delacqua F, Kirby J, Duda SN (2019). The REDCap consortium: building an international community of software platform partners. J Biomed Inform.

[47] Harris PA, Taylor R, Thielke R, Payne J, Gonzalez N, Conde JG (2009). Research Electronic Data Capture (REDCap)--a metadata-driven methodology and workflow process for providing translational research informatics support. J Biomed Inform.

[48] Kabat-Zinn J, Massion AO, Kristeller J, Peterson LG, Fletcher KE, Pbert L, Lenderking WR, Santorelli SF (1992). Effectiveness of a meditation-based stress reduction program in the treatment of anxiety disorders. Am J Psychiatry.

[49] Gómez Penedo JM, Berger T, Holtforth MG, Krieger T, Schröder J, Hohagen F, Meyer B, Moritz S, Klein JP (2020). The Working Alliance Inventory for guided Internet interventions (WAI-I). J Clin Psychol.

[50] Gimpel H, Huber J, Sarikaya S (2016). Customer satisfaction in digital service encounters: the role of media richness, social presence, and cultural distance.

[51] Gunawardena CN (1995). Social presence theory and implications of interaction and collaborative learning in computer conferencing. Int J Educ Telecomm.

[52] Kreijns K, Kirschner PA, Jochems W, van Buuren H (2007). Measuring perceived sociability of computer-supported collaborative learning environments. Comput Educ.

[53] Kreijns K, Kirschner PA, Jochems W, van Buuren H (2010). Measuring perceived social presence in distributed learning groups. Educ Inf Technol.

[54] Sicard V, O'Kane K, Brown O, Butterfield L, Kardish R, Choi E, Healey K, Silverberg ND, Smith A, Goldfield G, Saab B, Gray C, Goulet K, Anderson P, Mackie C, Roth S, Osmond MH, Zemek RL, Cairncross M, Ledoux AA Acceptability, usability, and credibility of a mindfulness-based digital therapeutic for pediatric concussion: a mixed-method study. JMIR Preprints.

[55] Hsieh HF, Shannon SE (2005). Three approaches to qualitative content analysis. Qual Health Res.

[56] Cho JY, Lee EH (2014). Reducing confusion about grounded theory and qualitative content analysis: similarities and differences. Qual Rep.

[57] Lattie EG, Stiles-Shields C, Graham AK (2022). An overview of and recommendations for more accessible digital mental health services. Nat Rev Psychol.

[58] Taylor CB, Fitzsimmons-Craft EE, Graham AK (2020). Digital technology can revolutionize mental health services delivery: the COVID-19 crisis as a catalyst for change. Int J Eat Disord.

[59] Li Q, Luximon Y, Zhang J (2023). The influence of anthropomorphic cues on patients' perceived anthropomorphism, social presence, trust building, and acceptance of health care conversational agents: within-subject web-based experiment. J Med Internet Res.

[60] Ivey AE, Ivey MB (1999). Intentional Interviewing and Counseling: Facilitating Client Development in a Multicultural Society.

[61] Oh CS, Bailenson JN, Welch GF (2018). A systematic review of social presence: definition, antecedents, and implications. Front Robot AI.

