# Correction: Development of Therapeutic Alliance and Social Presence in a Digital Intervention for Pediatric Concussion: Qualitative Exploratory Study

**DOI:** 10.2196/59722

**Published:** 2024-05-07

**Authors:** Kiarah M K O'Kane, Thalia Otamendi, Noah D Silverberg, Esther Choi, Veronik Sicard, Roger Zemek, Katherine Healey, Olivier Brown, Lauren Butterfield, Andra Smith, Gary Goldfield, Rachel Kardish, Bechara J Saab, Andrée-Anne Ledoux, Molly Cairncross

**Affiliations:** 1 Department of Psychology University of British Columbia Vancouver, BC Canada; 2 Department of Physical Therapy University of British Columbia Vancouver, BC Canada; 3 Children's Hospital of Eastern Ontario Research Institute Ottawa, ON Canada; 4 Department of Pediatrics and Emergency Medicine University of Ottawa Ottawa, ON Canada; 5 School of Psychology Faculty of Social Sciences University of Ottawa Ottawa, ON Canada; 6 Department of Neuroscience Carleton University Ottawa, ON Canada; 7 Mobio Interactive Singapore Singapore; 8 Department of Cellular and Molecular Medicine University of Ottawa Ottawa, ON Canada; 9 Department of Psychology Simon Fraser University Burnaby, BC Canada

In “Development of Therapeutic Alliance and Social Presence in a Digital Intervention for Pediatric Concussion: Qualitative Exploratory Study” (JMIR Form Res 2024 Mar 22:8:e49133) the authors noted several errors.

In the originally published article, the equal contribution note indicating that Andrée-Anne Ledoux and Molly Cairncross had contributed equally as co-senior authors was missed. In the corrected version, these authors are now listed as

Andrée-Anne Ledoux*; Molly Cairncross*

*these authors contributed equally

Then, in the originally published article, affiliation 5 was erroneously listed as

School of Psychology, Faculty of Social Sciences, University of Ottawa, Ottawa, BC, Canada

This has been corrected to

School of Psychology, Faculty of Social Sciences, University of Ottawa, Ottawa, ON, Canada

In addition, one author's name was listed as

Bechara Saab

This has been changed to

Bechara J Saab

The correction will appear in the online version of the paper on the JMIR Publications website on May 7, 2024, together with the publication of this correction notice. Because this was made after submission to PubMed, PubMed Central, and other full-text repositories, the corrected article has also been resubmitted to those repositories.

