# Supplementary figures and images for: Development of Therapeutic Alliance and Social Presence in a Digital Intervention for Pediatric Concussion: Qualitative Exploratory Study
Source: JMIR Form Res. 2024 Mar 22;8:e49133. doi: 10.2196/49133 (PMC10998177; doi:10.2196/49133)

## Appendix A

### Images of Intervention Content


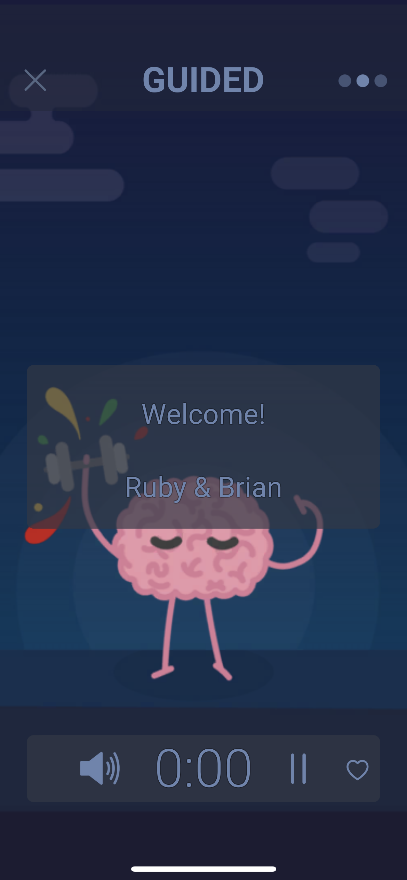

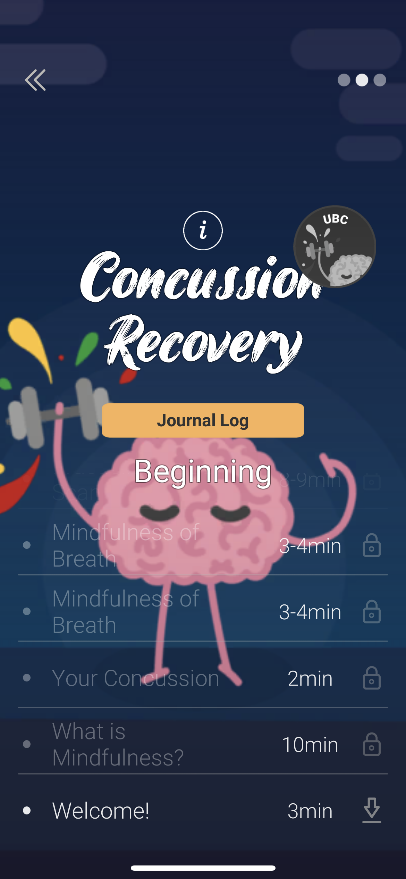


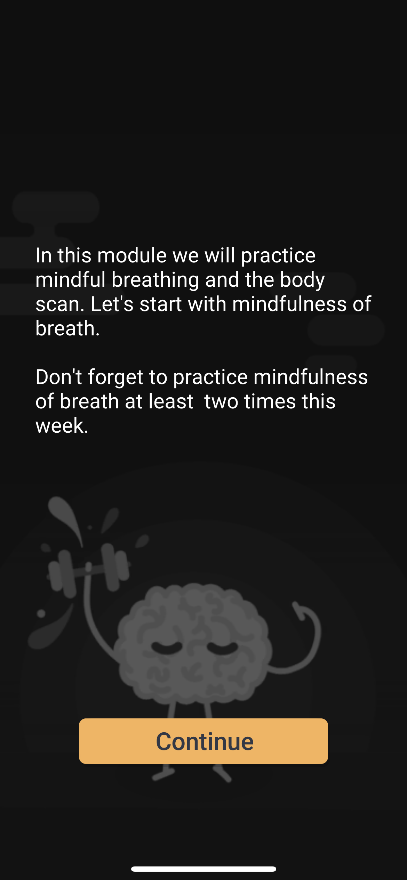

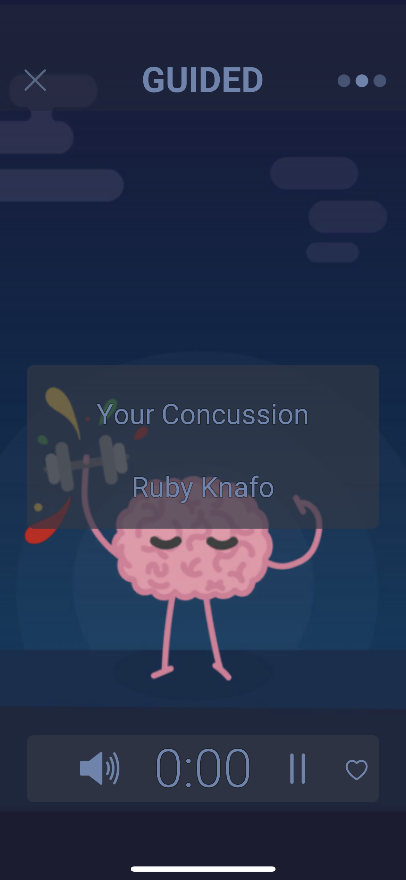

Supplement: Multimedia Appendix 1 [file formative_v8i1e49133_app1.docx]
