# Supplementary material for: Development of Therapeutic Alliance and Social Presence in a Digital Intervention for Pediatric Concussion: Qualitative Exploratory Study
Source: JMIR Form Res. 2024 Mar 22;8:e49133. doi: 10.2196/49133 (PMC10998177; doi:10.2196/49133)
Supplement: Multimedia Appendix 2 [file formative_v8i1e49133_app2.docx]

## **Appendix B**

### **Texting Protocol**

**How to use this protocol:**

The intended use of this manual is to provide guidance for those acting as a treatment coach in the MBI-4-mTBI study. Please read through this manual carefully and refer to it when texting participants. At the end of this document, there is a page on important considerations (page 9). Please review this page for general texting rules and guidance on wording before initiating text-communication with participant(s).

The document is organized by general order of protocol and includes texting scripts for possible issues that may arise or participant questions you may receive. Please begin your text-communication by sending the introductory text script below. As conversations are unique, there may be times where the provided script is not contextually appropriate. You make minor adjustments to text templates as appropriate, to fit the needs of the participant and the nuances of the conversation..

If anything is unclear or you come across a situation that the protocol does not cover, please email Molly (molly.cairncross@Ubc.ca) and she will provide further insight where needed!

**Introduction to text protocol and times/date for exchanges**

Hi there! My name is ____ and I’ll be your treatment coach for the next few weeks. As your coach, I will provide tech support and answer your questions about the study or material you are learning. I will check in with you each week to see if you have questions or concerns. If there is something I cannot help you with, I will try to find out where you can get the help. Please be aware that our texts may not be monitored constantly, so if you have an emergency of any kind, please immediately tell a parent or guardian, a doctor or medical professional, or call 911.

I wanted to ask when a good day of the week would be for short weekly check-ins. Please let me know which of these options work best for you: [weeknight #1], [weeknight #2], [weeknight #3], or [weekend day].

**Weekly Check In:**

Hi [insert participant name], this is [insert coach name], your treatment coach! I wanted to check in to see how your [X number] week using the app has been. What has your experience with the app been like this week?

- *If* ***they provide a specific issue they experienced****, follow protocol outlined below.*
- *If* ***they answer in very short sentences/very vaguely (e.g. “It was good.” Or “It was bad”)****, probe for more information.*
- “I’m glad to hear that it went well. What were some of the things you enjoyed about the study this week?
- “I’m sorry to hear that things were hard/not good this week. What was the worst part?”
- *Follow protocol outlined below for specific issues that participants mention.*

**Goodbye message after interaction is complete:**

Great work this week, [name]! Good luck with week [X]. Talk next [day of the week]!

**If participant does not reply after 24 hours:**

Hi [name]! I’m just following up with my previous text. I wanted to check in to see how your [X number] week using the app has been. I look forward to hearing from you!

**If participant does not reply after 48 hours:**

Hi [name]! I hope you’re doing well. I just wanted to let you know that this will be the last time I am contacting you this week, and will check in with you on the same day, same time, next week. However, if there is anything you want to discuss about this week, please let me know by simply replying to this text and I will try to support you in any way I can! Have a great week!

**How to respond to specific questions/comments:**

**Tech issues with application**

- START WITH: I’m sorry to hear that you are having trouble with [nature of issue e.g. “logging in”]. That can be frustrating! [Include blurb for the tech issues listed below].
  - Cannot log in (due to app, not wrong password)
    - Let’s try completely closing the app and restarting it as it may just be a startup glitch.
    - Did that work?
    - If no: Please contact Mobio tech support for further assistance as this seems to be an application issue.
  - Forgot password or username
    - If username: Your username will always be the email address you used to sign up for the app. Please try inputting your email address and password into the app!
    - If password: If you forget your password, you can reset it through Mobio! Please click “Reset Password” at the bottom of the sign-in panel. This will email you instructions on how to reset your password.
    - If the participant says that didn’t work: Hmm, I’m sorry that didn’t work for you. Please contact Mobio tech support for further assistance as this seems to be an application issue.
  - Slow to load?
    - Are you connected to a strong internet network? Please try moving closer to your internet modem, or moving to a place with better cellular connection as this may speed up the app and require less waiting time on your end.

If their wifi connection is weak:

- - - Your device may be connected to a weak internet service. Do you want to try turning OFF your wifi and connecting to mobile data instead?

If their data signal is weak:

- - - Your device may be connected to your mobile data rather than your home wifi. Sometimes, our data service can be weak and it can be faster to connect to an internet source. Do you want to try doing this?
  - Application crashes
    - Please try force-closing and restarting the app. If this does not work, please delete and reinstall the app from your application store as you can simply log back in to access all of your progress.
    - If the participant says that didn’t work: I’m sorry that didn’t work for you. Please contact Mobio tech support for further assistance as this seems to be an application issue.
  - Audio does not play
    - Your device audio may not be turned up high enough, or you may need to turn on your Ringer/Vibration function in order to play the sound. Sometimes, devices get confused with regards to outputs and need to be fiddled around with. You may also want to try using headphones instead to ensure a direct output!
    - If the participant says that didn’t work: Please try force-closing and restarting the app. If this does not work, please delete and reinstall the app from your application store as you can simply log back in to access all of your progress.
    - If the participant says that didn’t work: I’m sorry that didn’t work for you. Please contact Mobio tech support for further assistance as this seems to be an application issue.

**Issues with exercises**

- - Difficulty focusing on exercises
    - It is completely normal to have difficulties concentrating for long periods of time. Most people find it hard to pay attention to mindfulness exercises at first. If you are noticing your mind wandering - that is great - that is exactly what you should be doing! Notice when the mind wanders and bring your attention back when it does. The more you practice the easier it will become. Are you doing the exercises in a quiet space?
    - If no: This week, try to find a quiet spot free of distractions to do your exercises, if possible. This might make it easier to focus on your exercises!
      - *If they don’t have a quiet space, follow the protocol for “does not have a quiet space to practice the exercises” below.*
    - If yes: Great! Another thing you can try is to start with the shorter exercises first. Over time, you might find that it becomes easier to focus on the exercises for longer amounts of time.
  - Amount of screen time is difficult
    - It sounds like screen time has been tough for you, which is a common experience for people recovering from a concussion! I’m sorry that you are experiencing that. Do you find screen time hardest during the guided practices, the homework, or both?
    - If guided practices: One thing you can try this week is putting your phone beside you when you are doing guided practices, to just listen to the audio tracks, rather than looking directly at your screen.
    - If weekly homework: When doing your weekly homework, try taking breaks from your screen every few minutes. You can look away for thirty seconds, and then resume doing the homework.
      - If breaks do not help: It is completely okay if you do your journaling on a piece of paper, rather than in the app, if this helps best!
    - If both: This week, try putting your phone next to you during the guided practices, so you don’t have to look at your screen. When doing your weekly homework, try looking away from your screen every few minutes for thirty seconds.
  - Does not have a quiet space to practice the exercises
    - It can definitely be tough to find a quiet space all to yourself to complete your exercises! Where do you currently do your exercises?
    - If a common area, like a family room: Is there another quiet room you could go to when doing your exercises, like your bedroom?
      - If yes: This week, try doing the exercises in that room instead, and see if that helps!
      - If no: One thing you can try is speaking with your parents/siblings/whoever is noisy and letting them know that it is important that you have a quiet space when working on your exercises. Try picking a time together when they can give you some quiet time to work on your exercises.
        - If there is no quiet time: Another option to try out is to use headphones to block out background noise! Noise-cancelling headphones may be especially helpful for this.
  - Feels too busy to complete the exercises
    - It is completely normal to find it hard to fit something extra into an already busy day! I have some recommendations for what we can try to make this easier for you. I recommend using some of the shorter practices. Sometimes it can be hard to practice are certain times, what time of day do you normally do your mindfulness exercises?
    - If morning: Great! One thing to try is to incorporate your mindfulness exercises into routines that you already have. For example, if you have a morning routine that you do every day, add your mindfulness exercises to that routine, so they become part of something that you do every day. If you walk to school, this might mean that you do your mindful walking on your way!
    - If night: One thing to try is to avoid leaving your mindfulness exercises to right before you go to bed, as it can be tricky to find time for exercises when you are ready for bed and feeling tired! Instead, try picking a time to complete them during the daytime or early evening. You might want to try completing your exercises right before dinner.
      - If they do not want to practice earlier in the day: If you do want to practice at night, I recommend trying to incorporate your mindfulness exercises into routines that you already have. For example, you can try completing your exercises right before you brush your teeth at night!
    - If no specific time: One thing that may be helpful is to set aside a specific time each day for your mindfulness exercises, as consistency might be helpful for you! You can set an alarm for that time each day to complete your exercises.
  - Trouble staying still for the duration of the exercises
    - I’m sorry you are having trouble staying still during the exercises, that must be frustrating! However, it is completely normal to have trouble staying still for an entire exercise at first. I recommend starting out with the shorter exercises. As you get used to them, it may become easier to stay still for entire exercises. Then, you can work on longer exercises!
  - The exercises are boring
    - It can definitely be tricky to stay engaged with the exercises at first! One thing that might help to change things up for you is to do your exercises in a different spot each time you work on them. I also want to remind you that it is completely fine to feel bored and that you are practicing mindfulness by noticing that feeling of boredom! There will be a variety of exercises you will learn throughout this program, and you may like some more than others along the way.
  - The exercises are upsetting/causing negative feelings
    - I’m sorry to hear that the exercises are upsetting for you, that must be really tough! What do you find upsetting about the exercises?
    - If it is because they remind the participant about their symptoms/concussion: It is common to find reminders of your injury upsetting, and these feelings are completely normal. Over time, you may find that the exercises remind you of your injury less, and that they become less upsetting for you. First, could you tell me in a little more detail about what symptoms are coming up and causing these upset feelings?
      - If headache: Thank you for sharing that with me! I’m sorry that you are noticing headaches when completing the exercises. If you get “stuck” noticing the headache, maybe try putting your attention on another part of your body, maybe a part that feels neutral or good!
      - If dizziness: Thank you for sharing that with me! I’m sorry to hear that you have been noticing feelings of dizziness when completing the exercises. One thing you can try is completing the exercises with your eyes open. You can also try different exercises, maybe some feel more comfortable than others.
      - If nausea due to movement/walking: Thank you for sharing that with me! I’m sorry to hear that you have been noticing feelings of nausea when completing the exercises. Instead of walking, you can alternatively just stand and attend to the sensations in your feet. I would suggest just doing what you are most comfortable with!
- They don’t know: Sometimes we experience big emotions without knowing why, which is completely normal! It is totally okay to feel upset after your exercises and be unsure of why. One thing you can try is taking a few deep, slow breaths before you get started! This can be a helpful and relaxing exercise. Another thing that you can try is to do something fun after you complete your exercises, like having a treat or playing a game!
  - - They express suicidal thoughts/thoughts of harming themselves: [Follow Distress Management Plan document]

**Issues with** **homework:**

- - Falling asleep during mindfulness exercises
    - It can be hard to stay focused and alert during the mindfulness exercises. Sometimes, it can be especially hard if you are already feeling tired from a long day. Can I ask what time you tend to do your mindfulness exercises?
    - If they say evening-time:
      - One thing I would suggest you try is doing your mindfulness exercises earlier in the day rather than later, to avoid any fatigue from the day from interfering with your exercises.
    - If they say morning-time:
      - One thing I would suggest you try is doing your mindfulness exercises later in the day rather than earlier. Some people are more tired in the mornings than in the afternoons or evenings, and it may be worth trying out a different time in the day if you find yourself dozing off during your morning exercises!
  - Hard to remember to practice
    - It can be tricky to remember to practice mindfulness when it is something new to you! One thing you can try this week is to schedule in a time every day to work on the app. You can set an alarm on your phone to remind you. Set the alarm for a time when you are most likely to practice!
    - *Please draw on things the participant has mentioned they found helpful and use it as motivation!*
  - Not enough time
    - It can be hard to fit time for homework into an already busy day! One thing we can try together today is picking a specific time each day to work on your homework, to help make it a routine part of your day. Let’s brainstorm a time in the week to stick to for these exercises. *[Brainstorm a day/time that works for them.]*
    - Another thing you can try is doing your mindfulness practice early in the day. Sometimes it can be hard to do homework when you are feeling tired and ready for bed! Some days you may have more time to spend on this, and some days you may have less. This is totally okay!
  - Hard to concentrate for long periods of time
    - We hear this a lot! One thing you might try is starting with the shorter practices and move up to the longer practices. The more you practice the easier it will become.
  - Get frustrated
    - I can completely appreciate that the homework might be frustrating for you to complete. What do you find frustrating about it?
    - It is too much work: Some days and weeks may be busier than others because of school, family commitments, or other activities! It’s okay if some days you only practice for shorter periods of time. Doing a even a little bit is better than nothing!
    - It is confusing: Sometimes this stuff can be confusing! What do you find confusing?
      - If using the app is confusing: [see tech issues section]
      - If the content is confusing: [see psychoeducation content section]

**Psychoeducation Content:**

- Asking for clarity on a topic or confusion about a topic?
  - Great question! If you go back in the app to [insert name of audio relevant to question], Ruby explains that concept!

**Emergency text protocol:**

- If a participant says anything about suicidal thoughts immediately begin the distress management protocol and contact Dr. Molly Cairncross

**If a participant asked a question that is not outline in this protocol or I [the coach] do not know how to answer:**

- That is a great/understandable [question/comment/concern]. I do not have the answer to that [question/comment/concern]. I am going to contact Dr. Molly Cairncross - the researcher in charge and ask her. I will respond to you as soon as I can!
- *For tech issues that you don’t know how to solve, direct them towards Mobio’s tech support.* Please contact Mobio tech support for further assistance as this seems to be an application issue

**Important Considerations:**

- **Number of texts**
  - Should be concise - should be approx. 160 characters.
  - If we have a question for participants, the question should come at the END of the text.
  - Should be neutral and consistent across participants and interactions.
  - If participants do not respond to initial texts each week within 2 hours, please send a follow-up text.
    - If they do not reply for over 24 hours, please send a secondary text letting them know that this is their last point of contact for that week, and that you will reach out again next week.
- **Timing of texts**
  - Weekly texts should be sent on the same day and same time (as much as possible).
  - Please only contact participants on evenings or weekends (not during school). Unless the participant has a special circumstance/is not at school during the day.
  - Please give participants three weeknights to choose from to suit both participant & coach schedules.
- **Emoji use**
  - Best to keep minimal, as younger participants may interpret emojis differently than we do.
- **Abbreviation/slang use**
  - Best to keep minimal, as participants may interpret it differently than intended/may not understand the usage.
  - Is best to stay professional and neutral where possible.
  - (Ex. “lol”, “lmao”, “brb”, “kk”, “gtg”, “Jk”).
