# Supplementary material for: Development of Therapeutic Alliance and Social Presence in a Digital Intervention for Pediatric Concussion: Qualitative Exploratory Study
Source: JMIR Form Res. 2024 Mar 22;8:e49133. doi: 10.2196/49133 (PMC10998177; doi:10.2196/49133)
Supplement: Multimedia Appendix 3 [file formative_v8i1e49133_app3.docx]

## Appendix C

### Semi-Structured Interview

Introduction

Hi [name], my name is [name] and I am a member of the research team at [name of institution] that is studying the concussion app. We had scheduled this time to talk about the app you have been using for the past four weeks, is now still an okay time to chat?

This is an interview about your experience with the mindfulness app that you have used for the past few weeks. This means that I am going to ask some questions about your overall experience with the app, and also your experience with the mindfulness guides who guided your mindfulness practices. I might ask you to give me some examples as well. There are no right or wrong answers to any of these questions, I would just like to know about your experiences with the app, from your point of view. Keep in mind that you can stop this interview at any time, for any reason, and no one will be upset or be mad at you. Do you have questions about any of that?

We will be recording this interview so that I can review your responses at a later date. The study team will remove any information that could be used to identify you from any study documents, including the information you give me during this interview. The results from this study will be published in academic journals, but no information that can be used to identify you (e.g., name, date of birth) will be included. We will use a pseudonym, or a fake name, when we publish the results of these interviews. Also, if you say anything that might identify another person, like a friend or family member’s name, or the name of a location (e.g., school), we will also remove that from the recordings.

This interview will take between thirty to forty-five minutes. Do you have any questions for me at this point?

Interview

1. Before we talk about the app, I’d like to hear a little bit about what you’ve been doing lately. Are you in school? Considering the unusual circumstances this year, what has been your favourite activity?

- Follow-up with age-appropriate prompts/questions, such as what grade are you in? What do you like about school? What do you spend most of your time doing?

Now, I would like to ask you some questions about the mindfulness app that you used. Do you have any questions before we get started?

1. In general, how easy was it to use the app?

- Why was it easy/not easy?
- Prompts:
- Was it easy to download and open the app? Why or why not?
- Was it easy to open and use the mindfulness practices? Why or why not?
- Is there anything that you think would make the app easier to use?

1. Can you tell me three things that you like the most about using the app?

- Why do you like ____ about the app? *(For all three.)*
- Prompts:
- How does _____ make you feel?
- Is this aspect of the app really important to your overall experience of the app?

1. Can you tell me anything that you disliked about using the app?

- Why do you dislike ____ about the app? *(For all three.)*
- Prompts:
- How does _____ make you feel? Is this aspect of the app really important to your overall experience of the app?

1. If you could, how would you change the app?

- Why would you change that about the app?
- Do you think it is important that this is changed?

1. Have you found the app helpful? Why or why not?

- Prompts (if yes):
- What did it help with? (e.g., stress management, managing concussion symptoms, etc.)
- Is there a specific aspect of the app you find most helpful?
- Do you feel like using the app has helped you in any specific real-life situations?
- Prompts (if no):
- Is there a specific aspect of the app you find least helpful?
- Do you feel like using the app has made it harder to deal with any real-life situations?

1. At the beginning of the program, you wrote down goals into the app. What were they?

- Do you feel like the work you did in the app helped you reach your goals?
- Prompts (if yes):
- How did the app help you achieve your goals?
- How did it make you feel that the app was helping you work towards your goals?
- Prompts (if no):
- Why did the app not help you reach your goals?
- How did it make you feel that the app was not helping you work towards your goals?

1. Can you tell me about one thing that you have learned so far by using this app?

- Do you think learning _____ was important? Why or why not?
- How did you learn _____ through the app?

1. Did the app feel like it was designed to support what you specifically needed it for (such as for your concussion symptoms or mood)? Why or why not?

- Do you think it is important that the app feels like it was designed to support what you specifically need it for?
- Would you change anything to make it feel more/less like it supports what you specifically need it for?

1. Would you recommend this app to a friend who has experienced something similar to you? Why or why not?
2. Is there anything else that you would like to share about your experience using this app?

Okay, so now I want to ask you a few questions about the mindfulness guides, Ruby and Brian. In the app, Ruby talked about mindfulness and how it may be helpful for your concussion, and both Ruby and Brian led you through your mindfulness practices. Do you have any questions about that?

1. Can you tell me about your guides?

- Can you think of three words to describe your mindfulness guides, that is, three words to describe what they are like?
  - What made you feel that way about your guides?
  - Does this make you feel close to your guides, or distant from your guides?

1. What did you like about your guides?

- Do you think that it is important that your guides are like that?

1. What did you not like about your guides?

- Why did you not like that about your guides?

1. Do you feel like you got to know your mindfulness guides when they introduced themselves at the start of the program?

- Is there anything that you would have liked to know about them, that they did not say?
- Do you think that it is important that you get to know your mindfulness guides?
- Are there other things that could be done to help you feel like you got to know your guides?

1. Can you tell me about your connection with your mindfulness guides?

- Can you think of three words to describe that connection?
- Prompts:
- What made you feel that way?
- Did having/not having a connection with the guide impact your experience of the app?
- Is there anything you would have liked to change about your connection with your mindfulness guides?
- Prompts:
- Why would you have liked to change that?
- How important do you think that change would be?

1. Tell me what you liked or didn’t like about doing this program on an app. In other words, what did you like or not like about the fact that your mindfulness guides are not physically present with you when you do your mindfulness practices?

- Why do you like/not like that?
- Does it feel like your mindfulness guides are with you when you do your mindfulness practices? Why or why not?
- Prompts:
- How does that make you feel?
- Is it important to you that it feels like your mindfulness guides are with you?
- Do your mindfulness guides feel like real people? Why or why not?
- Prompts:
- Do their voices sound like those of real people?

1. Is there anything else that you would like to share about your experience with the guides?

- How important do you think your mindfulness guide is to how happy you are with the app? Why?

While you were using your mindfulness app, a member of the research team, your “coach,” texted you every week to talk about how your experience with the app was going.

1. Were you satisfied with the level of support that you received from your coach over text? Why or why not?

- Do you feel like your coach helped you work towards your goals? Why or why not?
- Prompts (if yes):
- What was the best part of your experience with your coach?
- Was there anything you wanted support with from your coach that you did not receive?
- Prompts (if no):
- What would have improved your experience with your coach?
- Was there anything you wanted support with from your coach that you did not receive?

1. Did it feel like your text coach was important to your experience using the app? Why or why not?
2. Is there anything else that you would like to share about your experience with the text coach?
